# Properties and Fabrication of PA66/Surface-Modified Multi-Walled Nanotubes Composite Fibers by Ball Milling and Melt-Spinning

**DOI:** 10.3390/polym10050547

**Published:** 2018-05-19

**Authors:** Tian Chen, Haihui Liu, Xuechen Wang, Hua Zhang, Xingxiang Zhang

**Affiliations:** 1 Tianjin Municipal Key Lab of Advanced Fiber and Energy Storage Technology, Tianjin Polytechnic University, Tianjin 300387, China; spfpolymer@sina.com (T.C.); wwwl-777@163.com (H.L.); xuechen-wang@126.com (X.W.); hua1210@126.com (H.Z.); 2 Key Lab of Advanced Textile Composite (Tianjin Polytechnic University), Ministry of Education, Tianjin 300387, China

**Keywords:** polyamide 66, surface modification, multi-walled carbon nanotube, composite fibers, physical mechanical property

## Abstract

PA66/surface-modified multi-walled carbon nanotubes (MWNTs) composite fibers with a better dispersion and a stronger interfacial interaction between MWNTs and polyamide 66 (PA66) matrix were fabricated via the method of ball milling and melt-spinning. The effects of unmodified (U-MWNTs), acid-modified (MWNTs-COOH) and sodium dodecyl benzenesulfonate-modified MWNTs (MWNTs-SDBS) on the physical mechanical and thermal properties of PA66 were investigated. The results show that, the surface modified nanotube can provide homogeneous dispersion and there is a strong interfacial bonding between PA66 and MWNTs-COOH. A homogeneous dispersion of MWNTs in PA66 matrices without agglomeration is obtained by a facile ball milling method, which can increase the utilization ratio of MWNTs, reduce the required amount of MWNTs and ultimately improve the mechanical properties at a lower filler loading. The tensile strength of composite fibers reaches a maximum which respectively improved by 27% and 24% than that of PA66 fibers, when the mass fraction of MWNTs-SDBS and MWNTs-COOH is 0.1%. It is helpful for decrease the producing cost of the composite fibers. Moreover, the incorporation of MWNTs into PA66 improves the crystallizing temperature, crystallinity and thermal stability. The research shows that a novel facile method is developed for the fabrication of polymer composite fiber.

## 1. Introduction

Polyamide-6, 6 (PA66) is a commercial semi-crystalline polymer. It is one of the most important high-performance engineering materials and it has been widely used for many applications, that is, sports-wear, carpet, netting twine and so forth, due to its excellent mechanical properties, wear resistant property, high thermal stability, processability and relatively low cost. However, its applications, that is, tire cord, conveyer belt, hambroline and so forth, are limited due to insufficient strength. To further improve its physical mechanical properties and wear resistance, some additives, such as glass fiber [1,2], carbon fiber [3], silica dioxide [4], carbon nanotubes (CNTs) [2,5,6,7,8,9,10,11,12] and graphene [13] and so forth, have been used in the research.

Multi-walled carbon nanotubes (MWNTs) is a kind of nanomaterial with excellent physical mechanical, electric and thermal properties. Its tensile strength and Young’s modulus is 11–63 GPa [14] and 270–950 GPa, respectively. It has attracted much attention as a material to heighten the strength of composite [2,5,6,7,8,9,10,11,12]. On the one hand, MWNTs is hard to disperse evenly in the polymer matrix (PM) due to its large ratio of length/diameter, high surface energy, van der Waals force inner and inter a single MWNT, which makes them entanglement seriously. On the other hand, the weak surface binding energy between polymer and CNTs is hard to transfer force from polymer to MWNTs, which lead to the result of MWNTs being pulled out under the shear stress [15]. The key issues to strength the composites are first how to disperse MWNTs evenly inside the PM and next how to enhance the biding energy. Many attempts, that is, in-situ polymerization [7,8], solution blended [16] and melting blended [9,17,18,19] method and so forth, have been tested recently. Meanwhile, noncovalent functional [20,21] and covalent functional [7,8,19,22] MWNTs were used to enhance the binding energy. However, there is still little information available as how to disperse CNTs evenly, low cost and quickly inside the polymer composite which hinders its industrial application.

Sodium dodecyl benzenesulfonate (SDBS) is a widely used ionic surfactant. The benzene ring can form π–π conjugate with the *sp*^2^ hybrid C=C bond on the surface of MWNTs and the polar sulfonate is helpful to disperse in polar water. SDBS was used to the liquid phase exfoliation graphene [23]. The exfoliated flakes are stabilized against reaggregation by a relatively large potential barrier, which originates in the Coulomb repulsion between surfactant-coated sheets. The application of SDBS as auxiliaries to help disperse MWNTs in PM has never been reported. Multi-walled carbon nanotubes (U-MWNTs), carboxylic MWNTs (MWNTs-COOH) and dodecyl benzenesulfonate-modified MWNTs (MWNTs-SDBS) were used as additives to modify PA66 composite fiber in this research. Ball milling and melt-spinning were also used to fabricate the composite fiber. The process is no solvent, low cost and suitable for industrial produce. Furthermore, the effects of these three additives and loadings on the properties of the composite fiber were further investigated.

## 2. Materials and Methods

### 2.1. Materials

PA66 powder was a product of BASF Inc., Ludwigshafen, Germany. It was vacuum-dried at 80 °C for 24 h before application. MWNTs and MWNTs-COOH with a mass purity of more than 95%, diameter less than 8 nm, lengths from 0.5 to 2 μm, were provided by Beijing Deke Daojin Co., Ltd. (Beijing, China). There are about 3.58% of surface carbon atoms of MWNTs are carboxyl groups in MWNTs-COOH which can from hydrogen bonds with PA66 chains. Sodium dodecylbenzenes sulfonate (SDBS, A.R.) and HCOOH (A.R.) were purchased from Tianjin Guangfu Reagents Company (Tianjin, China).

### 2.2. Fabrication of MWNTs-SDBS

The MWNTs (1 g) and SDBS (1 g) were dispersed in distilled water (200 mL) and then sonicated for 2 h. After that, the dispersed mixture was separated by using a Buchner filter and was washed repeatedly in distilled water. Finally, the mixture was dried in a vacuum oven at 80 °C for 12 h. MWNTs were modified with SDBS and named as MWNTs-SDBS.

### 2.3. Fabrication of Composite Powders

**Figure 1:**
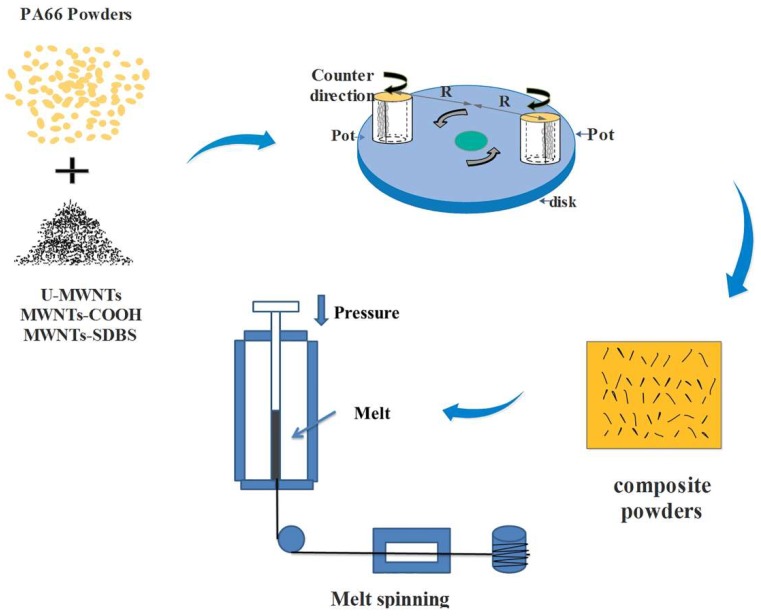
Schematic process of fabrication of PA66/multi-walled nanotubes (MWNTs) composite via ball milling and melt spinning process of composite fibers.

The MWNTs powders were milled with PA66 powders using a planetary ball mill (Chunlong Experimental Equipment Co., Ltd, Lianyungang, China). The diameters of the larger and the smaller quartz balls were approximately 10 and 6 mm, respectively. The quantity proportion of large quartz balls and small quartz balls was 1:6 and ball-to-powder mass ratio (BPR) was 2:1. The mass loadings of every U-MWNTs, MWNTs-COOH and MWNTs-SDBS samples in the composite powders were 0, 0.05, 0.08, 0.1 and 0.3 wt %, respectively. Grinding was performed as following: predetermined mass ratios of U-MWNTs, MWNTs-COOH, MWNTs-SDBS and PA66 powders were premixed in a beaker, then the two types of quartz balls were mixed and the mixture was placed into a cylindrical polyamide jar pot. The powder mixtures were milled for 3 h at a constant rotational speed of 100 rpm [12]. Afterwards, a series of PA66/U-MWNTs, PA66/MWNTs-SDBS and PA66/MWNTs-COOH composite powders were obtained after ball milling. PA66/MWNTs powders captured by balls during milling and MWNTs distributions during milling at various period of times and rotation rates are illustrated in Figure 1.

### 2.4. Melt-Spinning of Composite Fibers

All of the composite fibers were fabricated by a melt-spinning process with a homemade piston spinning machine by using the obtained powders. All of the powders were kept at 280–285 °C for 10 min to melt totally and then melt spun with a spinneret of 0.3 mm in diameter and a winding velocity of 20 rpm.

### 2.5. Characterization

The dispersibility of MWNTs in the PA66 matrix and surface morphology of the PA66/MWNTs composite fibers were observed by using a field emission scanning electronic microscope (FESEM; Hitachi S4800, Tokyo, Japan).

The FT-IR spectra of U-MWNTs, MWNTs-COOH and MWNTs-SDBS were obtained by a Bruker Vector 22 spectrometer (Bruker TERSOR37, Karlsruhe, Germany) in the range of 4000 to 500 cm^−1^. The resolution is 4 cm^−1^.

Apparent density test was performed based on GB/T 12496.1-1999. An accurate amount of 300 mg of the MWNT samples was weighed after being dried under vacuum at 80 °C for 24 h. The apparent volume was measured by using a cylinder (10 mL) and then the apparent density was calculated. For each apparent volume, the test was repeated 3 times and finally an average was calculated.

To observe the structure of MWNTs after ball milling, the composite powders (3 g) were dissolved into formic acid (200 mL) and then centrifuged at 8000 rpm. The collected MWNTs were re-dispersed in formic acid (200 mL) and the MWNTs were collected again. This procedure was repeated five times to remove PA66 completely. The final product was dried in a vacuum oven at 80 °C for 12 h. Transmission electron microscopy (TEM) was performed with the use of a Hitachi H-7650 electron microscope (Hitachi, Japan) which operated at an accelerating voltage of 100 kV. X-ray diffraction (XRD) and the azimuthal patterns were obtained using a D/MAX-2500 (Rigaku, Japan) (λ = 0.15418 nm). Micro-Raman mapping spectra (Horiba, Japan) was recorded on a Horiba Jobin Yvon Xplora confocal Raman microscope equipped with a 532 nm laser.

Thermal properties were measured using a differential scanning calorimeter (DSC, NETZSCH DSC 200 F3, Selb, Gemany). The determination temperature varied from 20 to 300 °C at a heating or cooling rate of 10 °C/min under a nitrogen atmosphere (0.2 MPa) and the retention period at both 20 and 300 °C was 3 min during the testing process. The DSC thermograms in the first cooling and second heating processes were recorded.

Thermal stability of the fibers was conducted on the fibers using a Thermogravimetric Analyzer (TG, NETZSCH STA409PC, Selb, Germany) from 20 to 1000 °C at a rate of 10 °C/min under a nitrogen atmosphere (0.2 MPa). The accuracy of mass measurement is 0.01 mg. In addition, the accuracy of temperature measurement is ±0.1 °C.

The mechanical properties of the fibers were obtained using a fiber tensile tester (LLY-06, Laizhou, China). The test used a fiber length of 10 mm at a rate of 10 mm/min. The results were obtained from the averaged results of 10 tests.

## 3. Results and Discussion

### Morphology and Structural of MWNTs

**Figure 2:**
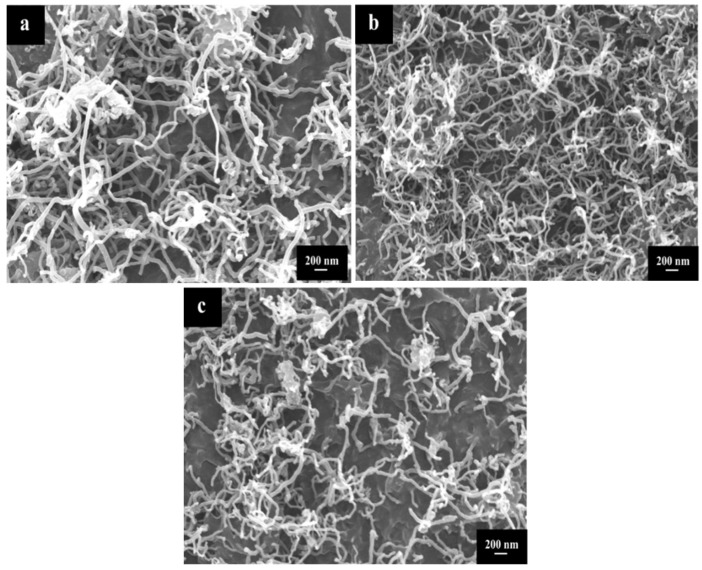
Scanning electron microscopy (SEM) micro-images of (**a**) U-MWNTs (**b**) MWNTs-COOH and (**c**) MWNTs-SDBS.

Figure 2 shows the typical SEM micro-images of U-MWNTs, MWNTs-COOH and MWNTs-SDBS. It is observed that three kinds of MWNTs are entangled and curled, however, their stacking morphologies are significantly different. It is observed that MWNTs-SDBS (2c) shows a looser stacking morphology compared with those of U-MWNTs (2a) and MWNTs-COOH (2b), which implies that SDBS treatment on the MWNTs can weaken the interaction force inner- and inter- MWNTs and therefore leads to a loose stacking morphology. However, acid treatment on the MWNTs will cause a strong interaction among MWNTs and thus will result in a compact stacking morphology of U-MWNTs [22].

**Figure 3:**
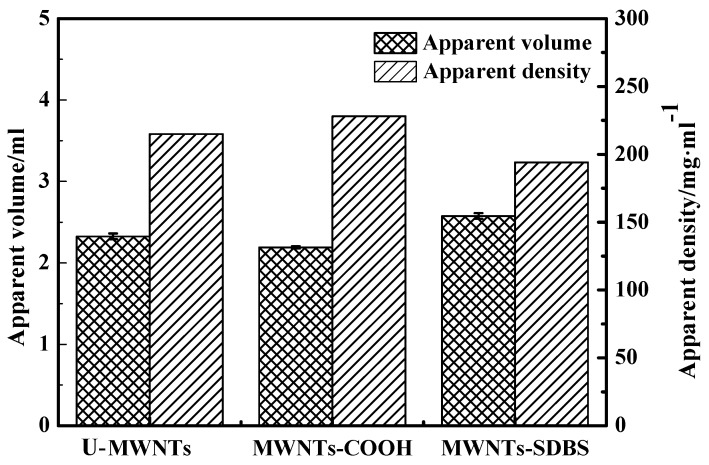
Apparent volume and apparent density of MWNTs-COOH, MWNTs and MWNTs-SDBS.

Figure 3 displays the apparent volume and apparent density of various MWNTs after various modifications. The apparent density is shown by the following sequence, MWNT-COOH > U-MWNTs > MWNTs-SDBS. The results indicate that acid treatment makes a more compact stacking of MWNTs, while the further SDBS modification may weaken the interaction force among MWNTs resulting in a loose stacking of MWNTs-SDBS. This is consistent with the results of SEM. The loose stacking morphology was more beneficial for MWNTs to disperse in PM.

**Figure 4:**
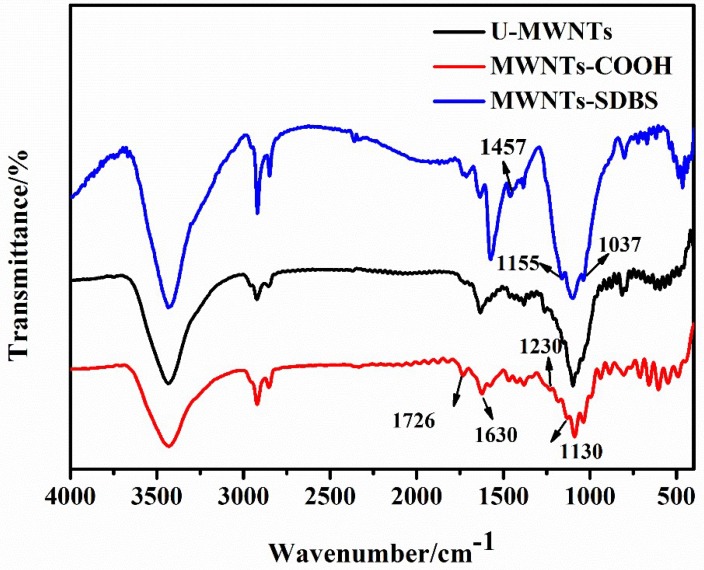
FTIR spectra of MWNTs-COOH, MWNTs and MWNTs-SDBS.

Figure 4 presents the FTIR spectra of U-MWNTs, MWNTs-COOH and MWNTs-SDBS. For the MWNTs-COOH, the absorption band located at 1726 cm^–1^ is attributed to the stretching vibrations of carboxyl groups. Another new band located at 1130 cm^–1^ is assigned to the C–O stretching vibrations. The difference between the characteristic peak in the spectra of U-MWNTs and MWNTs-COOH indicates that the surface of MWNTs has been successfully functionalized with carboxylic acid groups. In the spectra of U-MWNTs and MWNTs-SDBS, a new band at 1457 cm^–1^ is assigned to C=C in benzene rings, and 1155, 1037 cm^–1^ peaks are assigned to sulfonate group. The difference between the characteristic peak reveals that MWNTs was successfully treated with SDBS.

**Figure 5:**
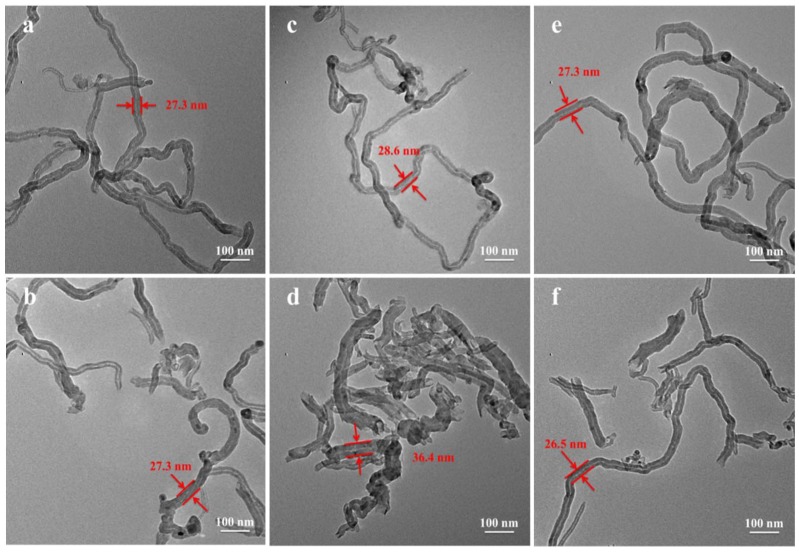
Transmission electron microscopy (TEM) micro-images of (**a**,**b**) U-MWNTs; (**c**,**d**) MWNTs-COOH and (**e**,**f**) MWNTs-SDBS before and after ball milling for 3 h and removal of PA66.

Figure 5 shows TEM micro-images of various MWNTs obtained after ball milling and removal of PA66. The length of the U-MWNTs and MWNTs-SDBS are slightly decreased and end tips are gradually opened. The diameters of the U-MWNTs and MWNTs-SDBS show almost no change after ball milling 3 h. However, the diameters of the MWNTs-COOH are significantly increased compared with the performance of U-MWNTs and MWNTs-SDBS, while the lengths of the MWNTs-COOH decreased most after ball milling 3 h, which are due to the strong interfacial interactions between PA66 and MWNTs-COOH and defects on the sidewalls of the MWNTs-COOH. These results are very similar to those results of solid-state shear milling (S^3^M) technique which can induce strong interfacial interactions between MWNTs and polyamide 6 [24]. The ball milling method can incorporate large amounts of N-groups onto the surface of CNTs [25]. We also proved that the strong interfacial interactions between PA6 and MWNTs-COOH were induced by the ball milling method in our previous studies [12]. Accordingly, we can infer that there is a strong interfacial interaction between PA66 and MWNTs-COOH, which makes it difficult to remove PA66 from MWNTs-COOH by the employed separation procedure, so the diameters of the MWNTs-COOH are significantly increased. In the process of preparing MWNTs-COOH, strong oxidizing agents (H_2_SO_4_ and HNO_3_) will produce oxygenated sites as defects on the sidewalls of the MWNTs [7] and the fracture of MWNTs generally occurs in the bending, kinking and other structural defects of nanotube walls in the ball milling process [12], so the lengths of the MWNTs-COOH decreased most.

**Figure 6:**
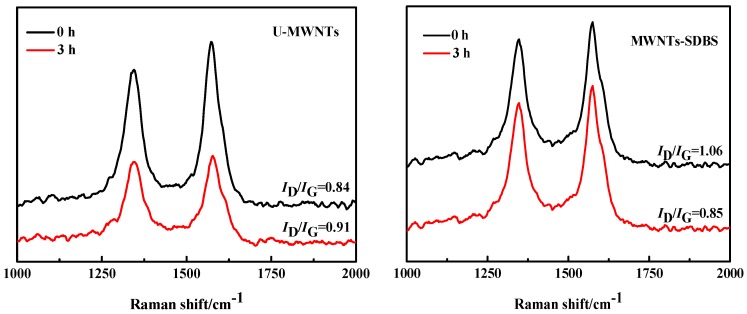

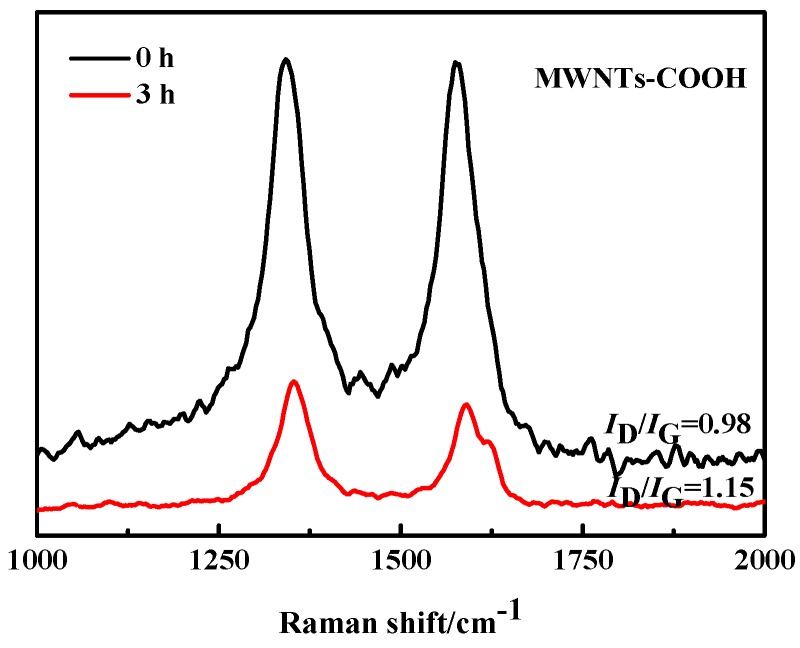
Raman spectra of MWNTs obtained before and after being milled for 3 h and the removal of PA66.

Figure 6 shows Raman spectroscopy of various MWNTs obtained after ball milling and the removal of PA66. For these three different kinds of MWNTs, it is possible to see evident bands at 1350 and 1580 cm^−1^, are in good agreement with the structures typically associated, respectively, to D band and G band. The ratio of the D band and G band intensity (*I*_D_/*I*_G_) is usually used to evaluate the defect concentration in carbon material. After ball milling 3 h, the U-MWNTs and MWNTs-COOH have a D band with increased intensity compared with that of the U-MWNTs and MWNTs-SDBS before ball milling, which can be attributed to the side defects after ball milling. These defects suggest that ball milling process should destroy structures of U-MWNTs and MWNTs-COOH. Conversely, the *I*_D_/*I*_G_ ratios for MWNTs-SDBS decrease after ball milling 3 h. This is probably because SDBS are adsorbed onto the surface of the MWNTs in the process of preparing MWNTs-SDBS. The side edge or surface defects of MWNTs are increased. However, the desorption of SDBS during ball milling or formic acid washing result in the decrease of the *I*_D_/*I*_G_ ratio of MWNTs-SDBS. Interestingly enough, the *I*_D_/*I*_G_ ratio of MWNTs-SDBS (0.85) after ball milling 3 h is similar to the *I*_D_/*I*_G_ ratio of pristine MWNTs (0.84). Thus, we infer that SDBS can protect the structure of MWNTs to some extent during ball milling process.

**Figure 7:**
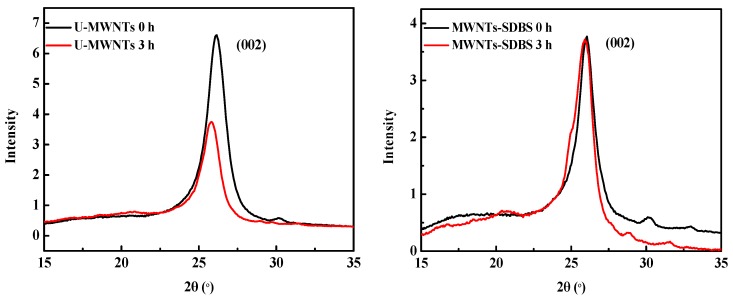

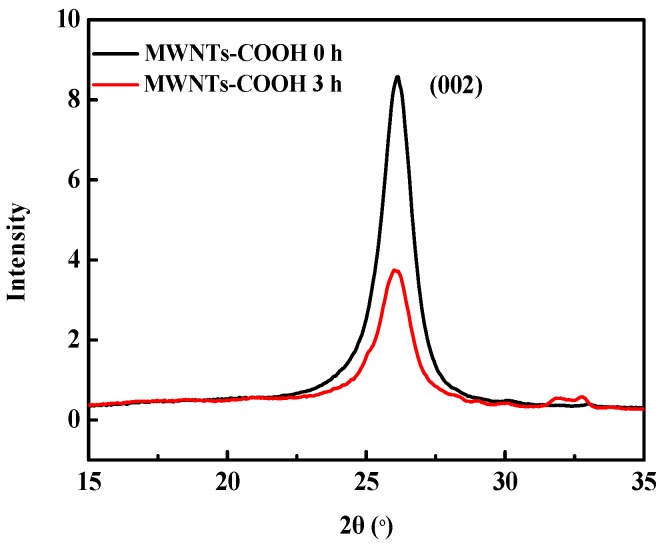
X-Ray diffraction (XRD) patterns of MWNTs obtained before and after being milled for 3 h and the removal of PA66.

The XRD patterns of MWNTs obtained after ball milling and removal of PA66 are shown in Figure 7. For the three different MWNTs, (002) diffraction peak of the MWNTs appears at 26° (2θ), which is attributed to the interlayer spacing of the carbon nanotube (d_002_). According to Bragg formula, the crystalline interplanar spacing is 0.34 nm. On the one hand, the peak position of the three different MWNTs shows no significant change after ball milling, thus the crystal structures of MWNTS are preserved. On the other hand, it is notable that the diffraction intensity of U-MWNTs and MWNTs-COOH decreases after ball milling. However, the diffraction intensity of the MWNTs-SDBS has no significant change after ball milling, which indicates that the crystallization property of the MWNTs-SDBS has been conserved during the ball milling process, which also further confirms that SDBS can effectively protect the integrity of MWNTs during ball milling process.

**Figure 8:**
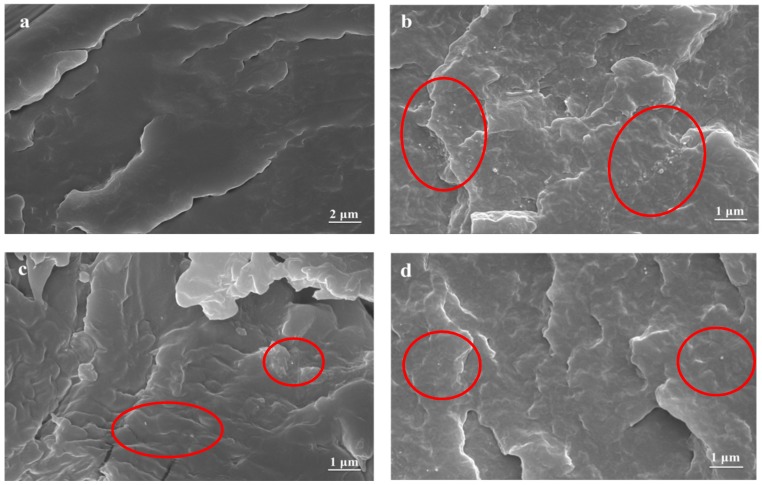
SEM micrographs of fractured surfaces of PA66 and the composite fibers ((**a**) PA66; (**b**) U-MWNTs; (**c**) MWNTs-COOH; (**d**) MWNTs-SDBS).

Figure 8 presents SEM micrographs of fractured surfaces of PA66/U-MWNTs, PA66/MWNTs-COOH and PA66/MWNTs-SDBS composite fibers with 0.3 wt % MWNTs, which were broken in a liquid nitrogen bath. The white dots or rods regions represent the broken ends of MWNTs that were stretched out of the PA66 matrix [17]. Both the MWNTs-COOH and MWNTs-SDBS were uniformly dispersed in PA66 matrix after ball milling 3 h; however, for the PA66/U-MWNT composite fibers, the U-MWNTs aggregates are dispersed nonuniformly in PA6 matrix and small aggregates can still be found. These data prove that the SDBS can weaken the interaction force among MWNTs and a better dispersion of the MWNTs-SDBS in the PA66 matrix can be achieved; moreover, there is a strong interfacial interaction between PA66 and MWNTs-COOH after ball milling (as proved by Figure 4). Hydrophobicity is improved in carboxylic functionalized MWNTs, so is, their dispersibility. In a word, surface-modified MWNTs can achieve a better dispersion in PA66 matrix as compared to U-MWNTs and they can even achieve a strong interfacial bonding with PA66 matrix.

**Figure 9:**
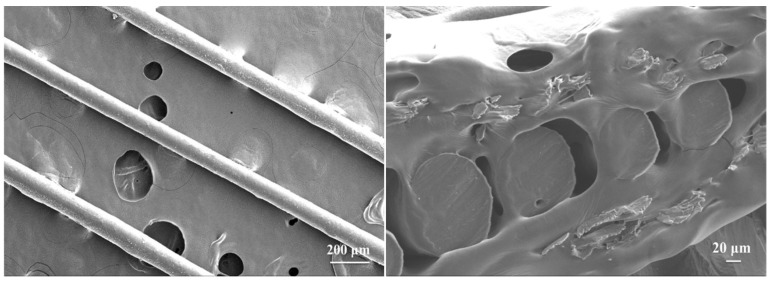
SEM micrographs of the surface and fracture surface of composite fibers.

Figure 9 presents SEM micrographs of the fibers surface and fracture surface of the composite fibers containing 0.3 wt % MWNTs-SDBS. The surface of the fiber is smooth. The diameter of the fiber, which is approximately 120 μm, is even. This performance further proves that the melting fluid of PA66/ MWNTs-SDBS can exhibit good fluidity, which reflects a good dispersion of MWNTs-SDBS in polyamide 66.

**Figure 10:**
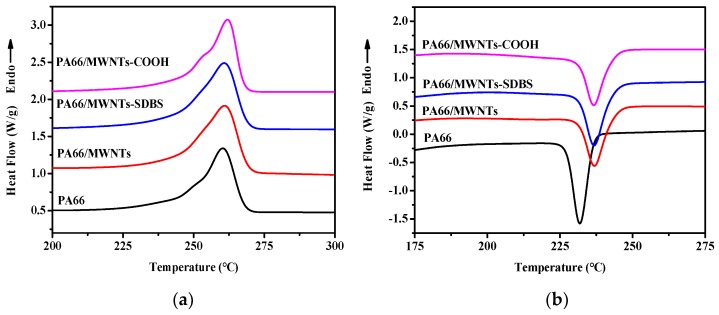
Differential scanning calorimeter (DSC) heating scans (**a**) and cooling scans (**b**) of the composite fibers.

The DSC curves of the composite fibers with 0.3 wt % additives are shown in Figure 10 and the results are also listed in Table 1. There is only one peak both in the heating scanning and cooling scanning. The melting peak temperature fluctuates at approximately 261 °C. In contrast, the crystallizing temperature increases about 5 °C after the addition of U-MWNTs, MWNTs-SDBS and MWNTs-COOH, respectively. Such a phenomenon can be explained by the function of MWNTs as a heterogeneous nucleating agent. The crystallinity of the composite fiber increases from 41.8% of PA66 to 47.7% of MWNTs-SDBS. Furthermore, it decreases to 45.3% of MWNTs-COOH. It is probably due to the dual function of MWNTs-COOH. It improves the crystallinity as a heterogeneous nucleating agent; and the chemical bonds between PA66 chain and MWNTs hinder the crystallization. The addition of MWNTs does not change the crystallographic form which is different from that in in-situ polymerization results of PA66 and MWNTs-COOH [7,8].

**Figure 11:**
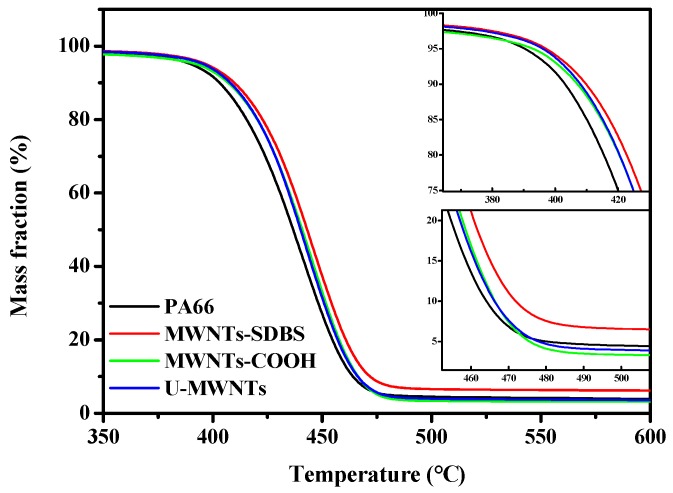
Thermogravimetric (TG) plots of the composite fibers.

The thermal stability of PA66 fiber and the composite fibers with 0.3 wt % additives are shown in Figure 11 and the results are listed in Table 2. The addition of MWNTs enhances the thermal stable temperature for 5.5–7.4 °C. The thermal stable temperature of the sample with 0.3 wt % MWNTs-SDBS is the highest which is assigned to the best dispersion of MWNTs in the PM. Such a phenomenon is significantly different from that in-situ polymerization resultant by using MWNTs-COOH and amino-MWNTs (MWNTs-NH_2_) as additives, respectively, in which the *T*i is 14–28.8 °C, which is lower than that of PA66 [7,8]. It is attributed to the PA66 chain length, which is bonded on MWNTs-COOH and MWNTs-NH_2_, is short. It implies that the PA66 chain length is not shortened seriously by the mill balling process.

**Figure 12:**
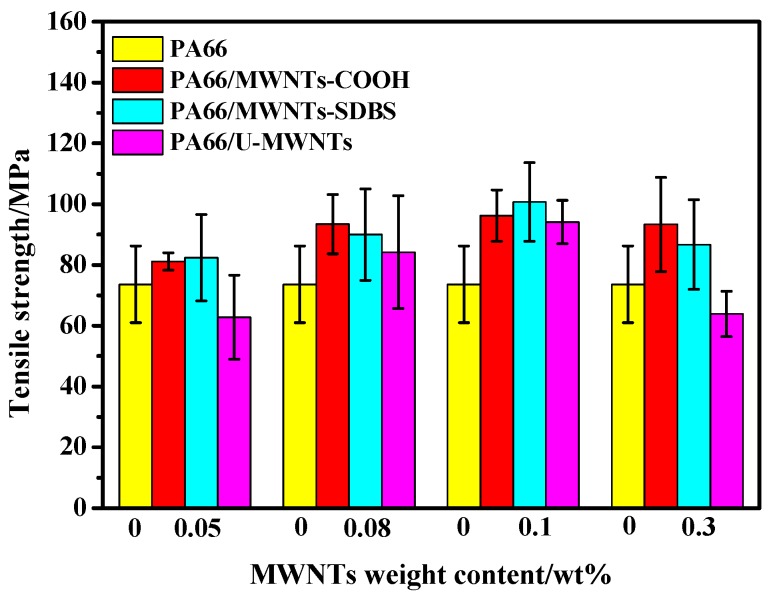
Tensile strength of the composite fibers.

Both the effect of various species of MWNTs and MWNTs loading on the tensile strength of composite fibers are plotted in Figure 12. Compared to the pure PA66 fibers, the tensile strength of composite fibers is greatly improved by the incorporation of MWNTs-SDBS and MWNTs-COOH, especially for loading with MWNTs-SDBS. From these facts, we further confirm that surface-modified MWNTs can achieve high dispersion and strong interfacial bonding with PA66 matrix. However, the addition of U-MWNTs has almost no reinforcing effect on PA66 and the tensile strength of PA66/U-MWNTs composite fibers was even lower than that of pure PA66 fibers with the addition of 0.05 and 0.3 wt % U-MWNTs. This is probably due to the not fully stretch which can be seen from the high strain. A maximum tensile strength for three kinds of composite fibers is achieved at the loading of 0.1 wt % MWNTs in PA66 and in general, using conventional wet and melt spinning, the maximum tensile strength and Young’s modulus of fibers were achieved at a loading of 0.5–1.0 wt % MWNTs in PA66 [7,8]. We believe that surface-modified MWNTs and ball milling method can achieve high dispersion and even stronger interfacial bonding with PA66 matrix, which can increase the utilization ratio of MWNTs, reduce the amount of MWNTs required and ultimately improve the mechanical properties at a low filler loading. Such a discovery is helpful for decrease the producing cost of PA66/MWNTs composite fibers. The tensile properties of PA66 and PA66/MWNTs composite fibers are summarized in Table 3. Obviously, the most effectively reinforced effect is shown in the following sequence, MWNTs-SDBS > MWNTs-COOH > U-MWNTs, which can be explained by the best dispersion of MWNTs-SDBS in PA66 matrix and the strong interfacial bonding between MWNTs-COOH and PA66. However, for U-MWNTs, small aggregates can still be found in PA66 matrix. These also confirm that, compared with U-MWNTs, surface-modified MWNTs exhibit more excellent modification.

## 4. Conclusions

The PA66/surface-modified MWNTs composite fibers were fabricated via ball milling and melt-spinning. The surface modified nanotube can provide homogeneous dispersion and there is a strong interfacial bonding between PA66 and MWNTs-COOH. A homogeneous dispersion of MWNTs in PA66 matrices without agglomeration is obtained by an easy ball milling method, which can increase the utilization ratio of MWNTs, reduce the required amount of MWNTs and ultimately improve the mechanical properties at a lower filler loading. The tensile strength of composite fibers reaches a maximum which is improved by 27% and 24% than that of PA66 fibers, respectively, when the mass fraction of MWNTs-SDBS and MWNTs-COOH are 0.1%. Moreover, the incorporation of MWNTs into PA66 improves the crystallizing temperature, crystallinity and thermal stability. The research shows that a novel facile method is developed for the fabrication of polymer composite fiber.

## Figures and Tables

**Table 1:** DSC melting and crystallization properties for PA66/MWNTs composite fibers.

Sample	*T*_m_/°C	Δ*H*_m_/J/g	*T*_c_/°C	*X*_c_/%
PA66	260.3	81.93	231.8	41.8
PA66/U-MWNTs	261.0	90.48	236.8	46.2
PA66/MWNTs-SDBS	260.8	93.49	236.7	47.7
PA66/MWNTs-COOH	262.0	88.76	236.6	45.3

**Table 2:** TG data of PA66/MWNTs composite fibers.

Sample	*T*_i_/°C	△*T*/°C*	*T*_10%_/°C	*T*_max_/°C
PA66	408.9	0	403.3	440.9
PA66/MWNTs	414.4	5.5	408.1	443.1
PA66/MWNTs-SDBS	416.3	7.4	409.9	445.4
PA66/MWNTs-COOH	414.5	5.6	406.8	446.2

* △*T* is the difference of the initial mass loss temperature of the composite fiber and the initial mass loss temperature of PA66 fiber.

**Table 3:** Mechanical properties of PA66/MWNTs composite fibers.

Sample	MWNTs loading (%)	Young’s modulus/GPa (Increase ratio)	Tensile strength/MPa (Increase ratio)	Strain/%
PA66	0	0.23	73.60	33
PA66/MWNTs-SDBS	0.05	0.30(26%)	82.40(11%)	27
	0.08	0.37(40%)	90.00(18%)	24
	0.1	0.33(32%)	100.70(27%)	30
	0.3	0.30(26%)	86.70(15%)	28
PA66/MWNTs-COOH	0.05	0.37(39%)	81.10(9%)	22
	0.08	0.38(40%)	93.40(21%)	25
	0.1	0.41(45%)	96.20(24%)	23
	0.3	0.47(52%)	93.30(21%)	20
PA66/U-MWNTs	0.05	0.13(−68%)	62.80(−17%)	47
	0.08	0.20(−13%)	84.20(13%)	42
	0.1	0.36(38%)	94.10(22%)	26
	0.3	0.27(15%)	63.90(−15%)	24
Ref 7(PA66/MWNT-COOH)	0.5	(65%)	(48%)	
Ref 16(PA66/MWNT-COOH)	1	(33%)	(49%)	
